# Factors affecting use of ballistics gelatin in laboratory studies of bacterial contamination in projectile wounds

**DOI:** 10.1186/s40779-018-0164-7

**Published:** 2018-05-25

**Authors:** Jessica J. Evans, Aaron Bost, Karim H. Muci-Küchler, Linda C. DeVeaux

**Affiliations:** 1 Biomedical Engineering Program, South Dakota School of Mines and Technology, 501 E. Saint Joseph St., Rapid City, SD 57701 USA; 2 Mechanical Engineering Department, South Dakota School of Mines and Technology, 501 E. Saint Joseph St., Rapid City, SD 57701 USA; 3 Department of Biology, New Mexico Institute of Mining and Technology, 801 Leroy Pl, Socorro, NM 87801 USA

**Keywords:** Ballistics gelatin, Wound, Contamination, Bacterial model

## Abstract

**Background:**

Ballistics gelatin is a common tissue surrogate used in bacterial contamination models for projectile wounds. Although these studies have demonstrated that bacteria are transferred from the surface of the gelatin to the wound track by a projectile, quantifiable results have been inconsistent and not repeatable in successive tests.

**Methods:**

In this study, five areas of a typical contamination model in which bacterial recovery or survival are affected were identified for optimization. The first was a contaminated “skin” surrogate, where the novel use of vacuum filtration of a bacterial culture and buffer onto filter paper was employed. The other possibly problematic areas of the bacterial distribution model included the determination of bacterial survival when the contamination model is dried, survival in solid and molten gelatin, and the effect of high-intensity lights used for recording high-speed video.

**Results:**

Vacuum filtration of bacteria and buffer resulted in a consistent bacterial distribution and recovery. The use of phosphate buffer M9 (pH 7) aided in neutralizing the ballistics gelatin and improving bacterial survival in solid gelatin. Additionally, the use of high-intensity lights to record high-speed video and the use of a 42^°^C water bath to melt the gelatin were found to be bactericidal for gram-positive and gram-negative bacteria.

**Conclusions:**

Multiple areas of a typical contamination model in which bacterial survival may be impeded were identified, and methods were proposed to improve survival in each area. These methods may be used to optimize the results of bacterial contamination models for medical applications, such as understanding the progression of infection in penetrating wounds and to identify possible sources of contamination for forensic purposes.

## Background

During armed conflicts, injuries to the extremities and subsequent infection are significant factors affecting a patient’s return of function and amputation risk [1]. Approximately 1600 extremity amputations were performed on military members from 2001 to 2013, and some were due to complications following infection [2]. Injuries resulting from debris ejected during the detonation of improvised explosive devices (IEDs) or from conventional bullets are of special interest. Knowledge of the microbial distribution throughout a wound track in a perforating projectile wound can aid in improving treatment protocols to reduce the risk of infection. Different authors have conducted controlled laboratory experiments to study the effect of parameters such as the projectile size, shape, and initial speed on the bacterial distribution in surrogate extremities shot with small caliber projectiles [1, 3–6]. Two important factors in such experiments that affect the quality of the experimental results are the biological tissue simulant used to make the target and the bacterial contamination model that represents the contamination source.

Synthetic ballistics gelatin (e.g., Perma-Gel®) and collagen gelatin are two commonly used biological tissue substitutes in terminal ballistics studies [7–9]. Penetration depths in the range of those of soft tissue have been demonstrated in both, particularly in the extremities such as the thigh muscle [7]. Both give reproducible results, are convenient to prepare, provide a humane alternative to using freshly killed or anesthetized animal models, and eliminate the requirements and difficulties involved with the use of cadavers. Because of their purity and homogeneity, they do not exactly replicate the characteristics and mechanical behavior of heterogeneous biological tissue, however, they are good options for initial testing and proof-of-concept modeling.

Despite the similarities between the two tissue simulants, there are notable differences between them that may affect specific applications. Clear synthetic gelatin is oil based, highly elastic, reusable, and easily visualized with high-speed video recording. However, it is composed primarily of mineral oil, which is poorly supportive of bacterial growth. Additionally, it melts at a very high, bactericidal temperature (121–132 °C) [10]. In contrast, collagen gelatin is translucent but not completely transparent, and may be used for high-speed video recording for visualization. Collagen gelatin must be prepared and used to precise concentration and temperature specifications, typically 10% weight by weight (*w*/w) and 4 °C or 20% w/w and 10 °C [11]. It is the medium commonly used as a soft tissue surrogate when studying projectile penetration events, and much of the reported research using collagen gelatin has focused on simulating wounds for both medical and forensic purposes [7, 12, 13]. A major advantage is that the recovery of contamination is easily accomplished due to its low melting temperature (~ 42 °C), making it a better option for bacterial distribution studies.

The current methods used in bacterial distribution studies include spreading bacteria onto surrogate “skin,” such as pig skin or sterile paper, mounting the “skin” onto a block of ballistics gelatin, and shooting a projectile through the contamination model and the target [3–6, 14–16]. Then, the “wound track” in the gelatin is resected and divided into segments. Finally, each segment is melted to release the contaminants and plated on media so that colonies can be counted. Although the results reported in the literature show that bacteria distributed on pig skin or paper do contaminate the entire wound track and projectile exit regions [4, 5, 14–17], the studies fail to demonstrate consistent quantifiable inoculation or repeatable results in parallel tests. Additionally, since human skin has an estimated 10^7^ bacterial cells per square centimeter [18], the accurate and consistent replication of this bacterial load in a model should yield more realistic results for skin contamination. It would also be helpful to change the cell density and composition to better simulate other sources of contamination, such as clothing or other environmental sources.

Another factor in contamination modeling is the contaminants themselves. Ideally, the bacterial inoculum must be representative of the source (e.g., skin) and, for safety reasons while conducting tests, non-pathogenic. For ease of use and to exclude other contamination sources that may be present in the laboratory environment, bacteria easily differentiated by color or antibiotic resistance should be used, allowing the elimination of those contaminants from the final count. A biosafety level-1 strain of *Staphylococcus epidermidis* is both representative of normal skin flora and non-pathogenic; alternatively, a lab strain of *Escherichia coli* K12 is non-pathogenic and easily transformed with plasmids, or small pieces of circular DNA, that can confer selectable colorimetric and/or antibiotic resistance characteristics. These plasmids are commercially available, diverse, and inexpensive. Other antibiotic resistance markers (e.g., chloramphenicol, kanamycin, streptomycin, etc.) or colorimetric proteins, such as green fluorescent protein (pGFP) and red fluorescent protein (pmCherry), may be used instead according to the research needs.

Establishing a consistent method of producing a microbial contamination model would be useful for bacterial distribution studies dealing with civilian and military medical and forensic applications. Contamination models may also be helpful for identifying the source of infection whether it is environmental or clinical. A modeling method aimed at providing a consistent contamination load and maximizing the recovery and viability of the bacterial contamination in the surrogate wound track is proposed in this study.

The model considered used vacuum filtration onto a piece of filter paper, allowing for a good estimate of the number of bacteria entering the wound versus the amount recovered, as well as consistent and homogeneous bacterial recovery. The effect of typical parameters of bacterial distribution experiments involving ballistics gelatin targets, such as the use of high-intensity lights for high-speed video recording, were tested against the bacteria selected for the contamination model. The adjustment of the pH of the ballistics gelatin with microbiologically relevant phosphate buffers to make it more appropriate for bacterial growth was also explored.

## Methods

**Fig. 1:**
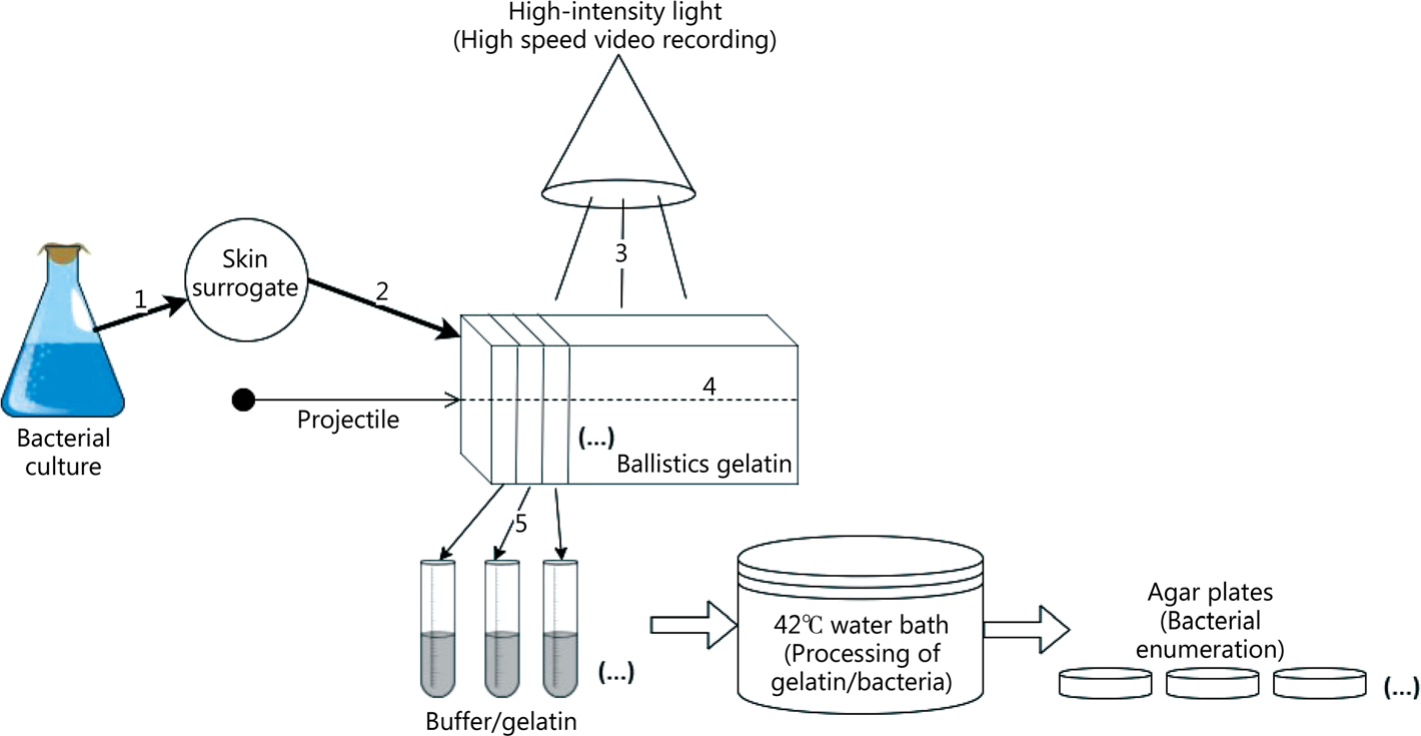
Schematic of a typical bacterial distribution study using ballistics gelatin as a tissue surrogate, and the potential complicating factors for consistent bacterial recovery

As shown in the schematic in Fig. 1, five different experiments were used to assess the suitability and performance of the proposed contamination model for bacterial distribution studies involving ballistics gelatin targets shot with small caliber projectiles. The first experiment focused on a determination of the consistency of the bacterial distribution and the recovery of the bacteria from the skin surrogate. The second considered how drying affected the bacterial recovery from the skin surrogate. The third evaluated the effect of the high-intensity lights used to record the high-speed video on bacterial survival. The last two dealt with bacterial survival once delivered into the ballistics gelatin target. The first considered how the bacteria tolerated being inside the solid gelatin, which is representative of the scenario before the permanent cavity is processed. The second assessed the effect of the procedure used to recover the bacteria from the surrogate wound on bacterial survival, which involved melting samples of ballistic gelatin containing the bacterial contamination in a buffer solution. Statistical significance was indicated at *P* < 0.05 and was calculated via a two-way ANOVA and a post-hoc Tukey USD test (R ×64, Version 3.4.2). The “Fraction Survival” was determined by dividing the survival at each time point by the corresponding survival at time zero.

This section presents the materials and methods used for the experiments mentioned above. First, materials and methods common to several experiments are considered. Then, information specific to each experiment is provided. All chemicals and consumable materials were obtained through Fisher Scientific (Waltham, Massachusetts, USA) unless specifically stated.

Two different types of bacteria were used: *Escherichia coli* strain DH5α containing the plasmid pUC19, which provides constitutive expression of ampicillin resistance (*amp*^*R*^) and inducible β-galactosidase (*lacZα*), and a biosafety level-1 strain of *Staphylococcus epidermidis,* which was obtained from Carolina Biological (Item # - 155,557, Burlington, North Carolina, USA). *E. coli* was grown in Miller LB broth with 100 μg/ml ampicillin at 37 °C with shaking for 24 h or until the stationary growth phase [i.e., ~ 1 × 10^9^ colony forming units (CFU) per milliliter] was reached. Cells were plated for colony visualization onto Miller LB containing 2% agar, 100 μg/ml ampicillin, 10 μg/ml Isopropyl β-D-1-thiogalactopyranoside(IPTG), and 20 μg/ml X-gal (a blue colorimetric lactose analog), and incubated at 37 °C for 24 h or until colonies were visible. *S. epidermidis* was grown in nutrient broth at 37 °C with shaking for 24 h. Cells were plated for colony visualization on solid media containing 2% agar.

Ballistics gelatin (VYSE® Professional Grade Ballistic Gelatin, Custom Collagen, Addison, IL) was prepared in accordance with standard methods at a gelatin concentration of 11% weight by weight (*w*/*w*) [11, 19]. The characteristics of the water and buffers used for dissolving the gelatin are presented in Table 1. Buffers were prepared with laboratory reverse osmosis (RO) water, unless stated otherwise.

**Table 1:** The properties of the solutions utilized to prepare the ballistics gelatin, including the buffer pH and the pH of the solid ballistics gelatin and the gelatin in buffer

Solution	pH buffer	pH gelatin	pH buffer/gelatin	Buffer composition
RO water	4.6	4.8	N/A	N/A
PBS	7.0	4.8	6.5	137 mmol/L NaCl, 2.7 mmol/L KCl, 10 mmol/L Na_2_HPO_4_, 1,8 mmol/L KH_2_PO_4_
M9	7.0	6.5	7.0	22 mmol/L KH_2_PO_4_, 42 mmol/L Na_2_HPO_4_, 86 mmol/L NaCl
M63	7.0	6.5	N/A	100 mmol/L KH_2_PO_4_, 15 mmol/L (NH_4_)_2_SO_4_, 1 mmol/L MgSO_4_, 1.7 μmol/L FeSO_4_
Sodium phosphate pH 8	8.0	7.3	7.8	93 mmol/L Na_2_HPO_4_, 7 mmol/L NaH_2_PO_4_,

N/A. not available RO water. Reverse osmosis; PBS. Phosphate-buffered saline

Considerations for standard bacterial distribution ballistics studies: 1) Consistent bacterial distribution and reproducible recovery, 2) The effect of drying the skin surrogate and recovery of bacteria, 3) The effect of high-intensity lights on bacterial survival, 4) Survival of bacteria in solid gelatin, and 5) Survival of bacteria in buffer/gelatin during sample processing.

### Bacterial distribution and recovery on filter paper

**Fig. 2:**
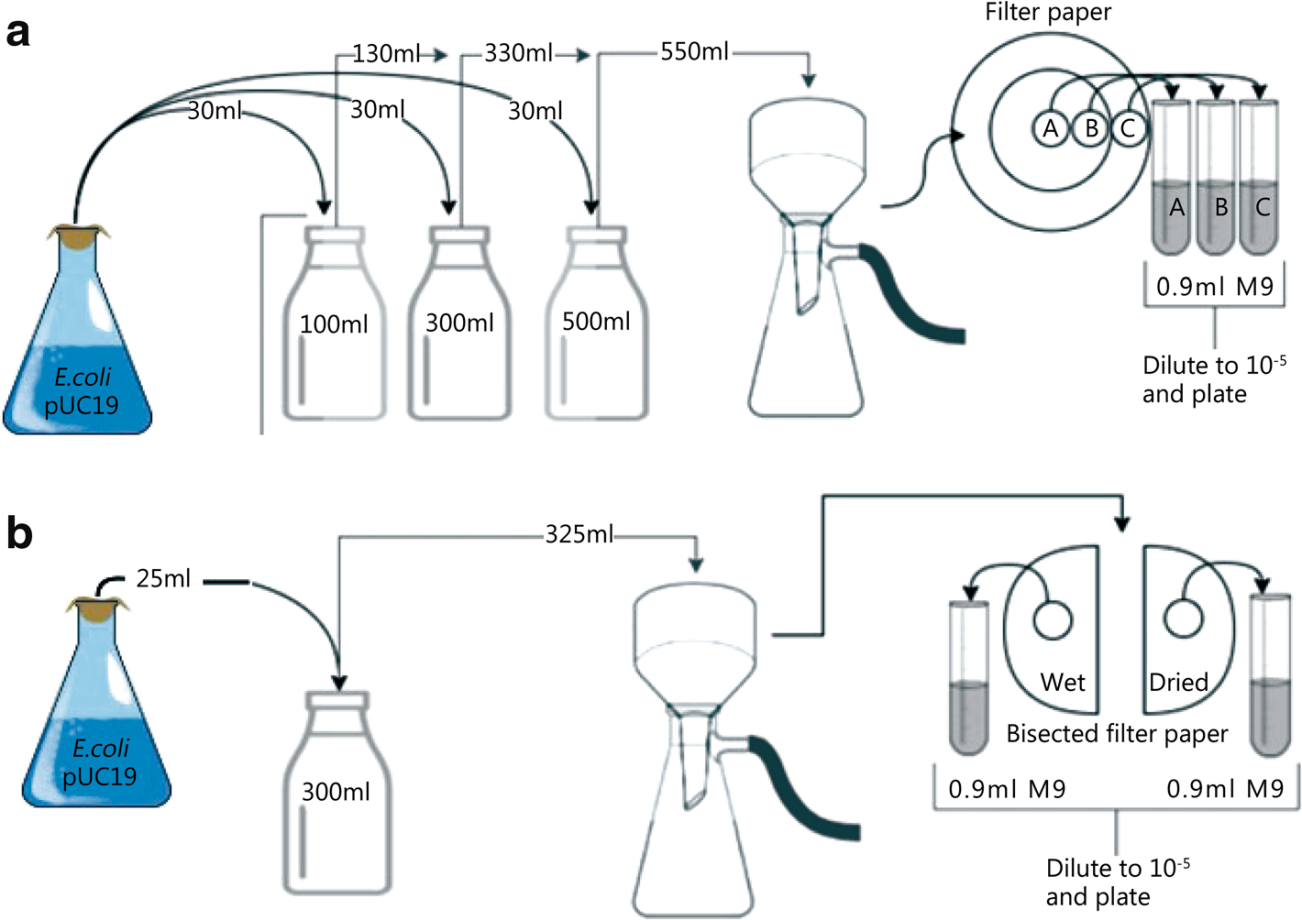
Schematic of filter paper distribution and recovery tests. Figure **a** represents the tests for the consistency of the bacterial distribution on the filter paper using different volumes of buffer diluent, while Figure **b** represents the tests of the effect of drying the filter paper on the bacterial recovery

Thirty microliters of a stationary phase bacterial culture were diluted in 100, 300, and 500 ml of sterile M9 buffer (Table 1) and evenly mixed. Each suspension was vacuum-filtered onto a 90 mm Millipore (Billerica, Massachusetts, USA) Express Plus® Membrane (0.22 μm pore size) using a glass microanalysis filter holder assembly connected to a vacuum pump. Each filtration experiment was performed in duplicate. Three separate zones of the filter were sampled (Fig. 2a) using a sterile 12 mm Acu-Punch® biopsy punch. Each filter paper punch was placed into 0.9 ml of sterile buffer, vortexed for 5 s to dislodge the cells, then allowed to incubate at room temperature for 30 min and vortexed intermittently. Each sample was vortexed for five seconds to distribute the cells, then serially diluted to 10^− 5^ in buffer. To visualize cells, 0.01 ml of each dilution was placed onto a square on a 6×6 gridded plate of solid medium.

The concentration of the recovered bacteria was divided by the total area of the biopsy punch (1.13 cm^2^) and compared to the amount loaded, which was determined based on the concentration and volume of the culture divided by the loaded area of the filter paper (47.78 cm^2^).

### Recovery from filter paper after drying

To test the effect of drying the filter paper loaded with bacteria before use, 25 ml of culture was diluted into 300 ml of sterile M9, and filtered in duplicate as shown in Fig. 2b. The filters were bisected aseptically. One half was sampled and processed as described in the experiment shown in the *Bacterial distribution and recovery on filter paper* section (Fig. 2a). The other half was placed into a covered 100 mm petri plate and incubated in a 37 °C incubator for one hour prior to sampling and processing.

### High-intensity light exposure

Ballistics gelatin was poured into four 150 mm×15 mm petri plates and a 33 cm×23 cm×5 cm metal pan, and allowed to set at 4 °C for 48 h prior to testing. Thirty milliliters of a stationary phase *E. coli* culture was diluted in 300 ml of M9 buffer and filtered as described in *Bacterial distribution and recovery on filter paper* section. Each of the four filters was placed on a petri plate filled with gelatin. The two control filters were not exposed to high-intensity light. The plates for the light exposure treatment were placed on top of the gelatin in the metal pan to keep the temperature of the gelatin in the plates as consistent as possible. Four ARRILUX Pocket PAR 200-W lamps (ARRI AG, Munich, Germany) were spaced evenly around the plates approximately 24 in. from the filter paper. At 0, 5, 10 and 15 min, the filters were sampled and processed as in the experiments described in sections of *Recovery from filter paper after drying* and *Recovery from filter paper after drying* (Fig. 2). Plating was done in triplicate.

### Survival in solid gelatin

To test the survival of the cells immediately after deposition into the gelatin and prior to recovery, 5 ml of ballistics gelatin prepared in an appropriate buffer (Table 1) was poured into 15 ml Falcon™ tubes and allowed to solidify at 4 °C for 48 h. Prior to the experiment, the gelatin was melted in a 42 °C water bath. At time zero, 0.1 ml of a saturated bacterial culture of either *E. coli* or *Staphylococcus epidermidis*, was combined with 5 ml of melted gelatin. The mixture was vortexed for 5 s, and 0.1 ml was immediately removed for diluting and plating. Each tube was then placed at 4 °C for one hour to allow the gelatin to solidify. At 60 min, the tubes were placed into a 42 °C water bath until the gelatin had just re-melted, then vortexed for 5 s. A 0.1 ml sample of the gelatin solution was then diluted and plated in triplicate.

### Gelatin and buffer survival

The ballistics gelatin was prepared as before using an appropriate buffer solution (Table 1) and poured to fill 150 mm×15 mm petri plates. A 12 mm biopsy punch of ballistics gelatin from the petri plates (approximately 1 ml) was placed into 5 ml of an appropriate buffer (the RO water gelatin was diluted in PBS) with 0.1 ml of bacteria in the stationary growth phase, then the tubes were placed in a 42 °C water bath to melt the gelatin. As soon as the gelatin was visibly melted, the tubes were vortexed, and 0.1 ml was removed for dilution and plating. This was repeated every 10 min for 40 min, and the plating was done in triplicate.

## Results

### Bacterial distribution in the vacuum filtration tests

While previous contamination models reported in the literature were adequate to demonstrate the post-penetration presence of bacteria throughout a wound track, the mode of inoculation of the skin surrogate made the bacterial quantity and homogeneity at the location where the projectile hit impossible to accurately predict. Controlling the concentration and bacterial distribution on the surface would allow a more accurate estimation of the number of bacteria that entered the wound. Vacuum filtration provides an excellent solution to this problem. A 90 mm Millipore filter paper with a 0.22 μm pore size was chosen as the skin surrogate based on two major considerations. First, the 0.22 μm pore size is used in microbiological applications for the sterilization of liquids; bacteria are too large to pass through and would therefore remain on the filter paper without any loss into the filtrate. Second, the large surface area of the paper allowed for a margin of imprecision in the impact location of the projectile.

**Fig. 3:**
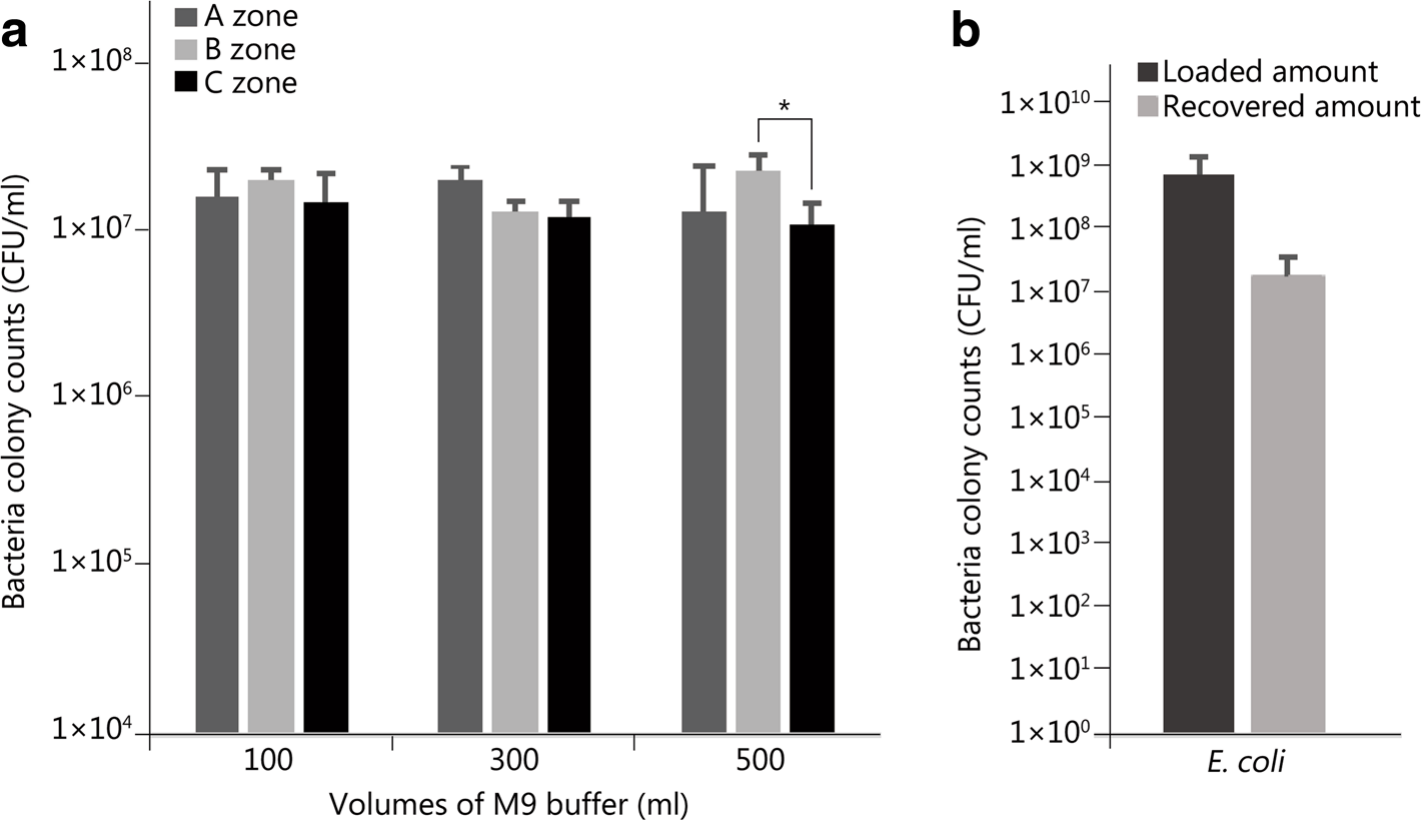
Recovery of *E. coli* from the filter paper. **a** Bacterial recovery from the filtration of three different volumes of M9 buffer combined with 30 ml of the *E. coli* culture. A, B, and C correspond to previously defined filter zones (See Fig. 2a). *. Only zones B and C of the 500 ml recovery show a significant difference of recovery (*P* = 0.03); **b**) Average recovery of *E. coli* from filter paper vs. the amount loaded. The error bars represent the standard deviation

To ascertain if a homogenous distribution of culture could be achieved via vacuum filtration, an experiment in which samples were taken from multiple areas of the 90 mm filter following filtration (Fig. 2a) was carried out. After counting the bacterial colonies that grew and comparing the numbers from the different filter zones, A, B and C (Fig. 3a), two conclusions were drawn. First, the volume of buffer had no significant effect on the recovery from the filter paper. Since each bottle contained the same number of bacteria, the number loaded onto each filter paper was the same. Second, the bacterial distribution was homogenous, as evidenced by consistent recovery across the filter.

### Reproducibility of recovery

To confirm the reproducibility of bacterial recovery from the filter paper, a total of 58 filter papers were loaded at various times, samples were taken from each, and the recovery was assessed. The recovery was consistent at approximately 2% of the bacterial load across all tests, including the previous one (Fig. 3b).

### Effect of drying on recovery

**Fig. 4:**
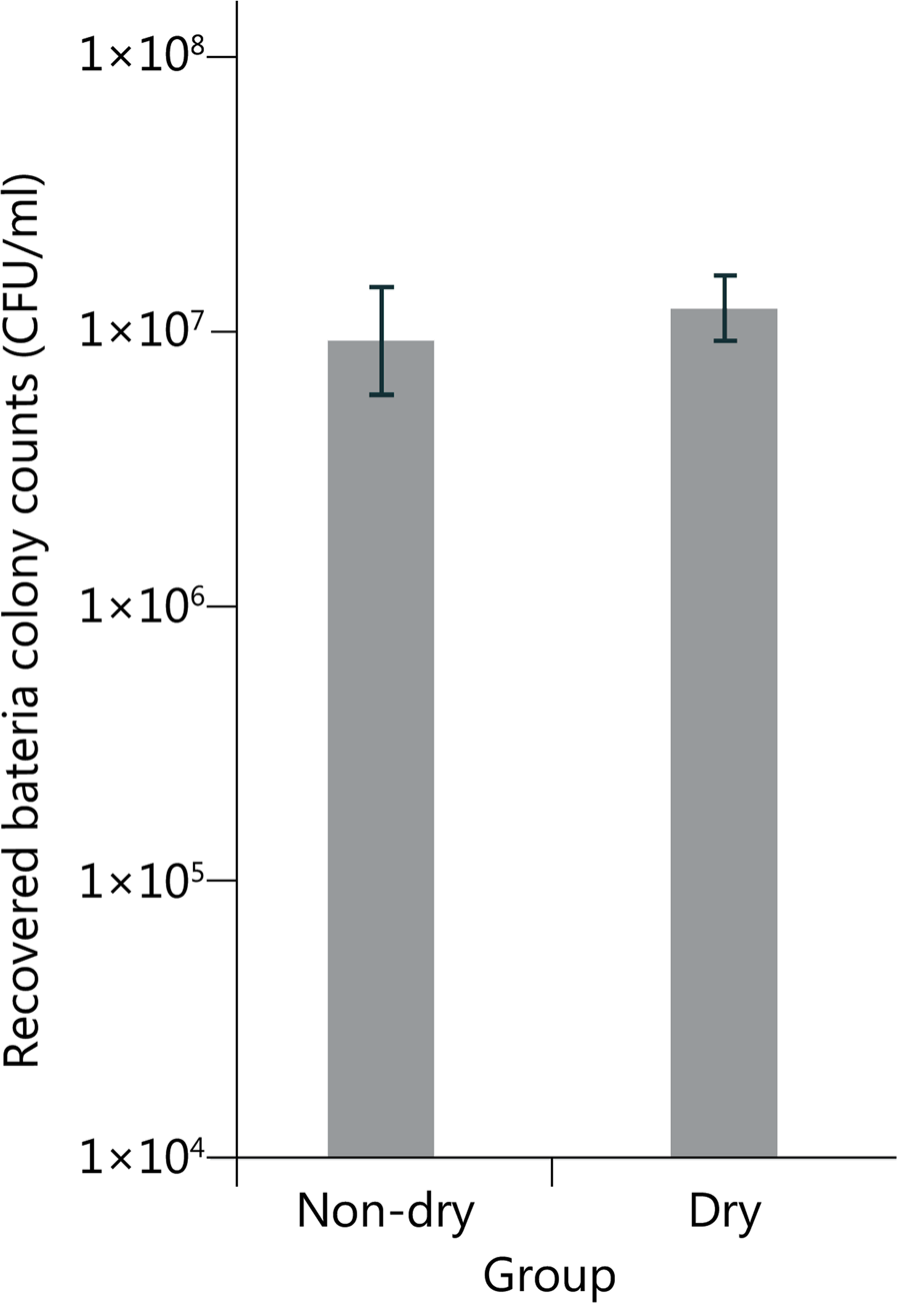
Bacterial recovery from the filtration of 300 ml of M9 buffer and 25 ml *E. coli* culture. Half of the filter was immediately processed and the other half was dried for one hour at 37 *°*C prior to processing. No significant difference was found between the two (*P* = 0.49). Error bars represent the standard deviation

In previous studies, the liquid culture applied to the surface of the paper/skin was allowed to dry before being placed in front of the ballistics gelatin target and shot, however, drying may impact bacterial adherence and subsequent recovery. To establish whether drying affected the viability or recoverability of *E. coli* from the filter paper, a sample of filter paper was dried, and the recovery was determined (Fig. 2b). There was no significant difference in recovery between the wet and dried filters (Fig. 4).

### Survival of bacteria during high-intensity light exposure

**Fig. 5:**
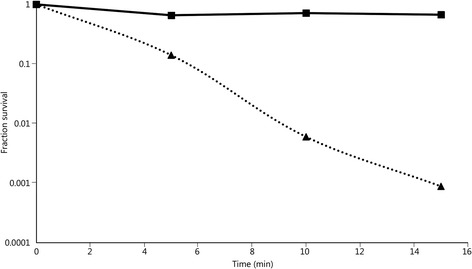
Fraction of survival of *E. coli* during high-intensity light exposure (dotted line; triangle) or unexposed (solid line; square) compared to time zero, The difference in the survival between the exposed and unexposed *E. coli* was significant (*P* = 0.01)

A high-speed video recording requires the use of high-intensity lights in close proximity to the target. These lights concentrate heat and UV radiation onto the filter paper containing the bacteria, which can be bactericidal*.* A test involving only the high-intensity lights and filter paper on gelatin was carried out to measure the survival of *E. coli* under these conditions. The cells on the filter paper were exposed to the high-intensity lights to simulate the time that a filter might be exposed during an actual bacterial distribution experiment, and cells were assayed every 5 min. Even during the first 5 min, there was a significant decrease in cell survival, and survival decreased by approximately 10-fold every 5 min (Fig. 5).

### Survival of bacteria in solid gelatin

**Fig. 6:**
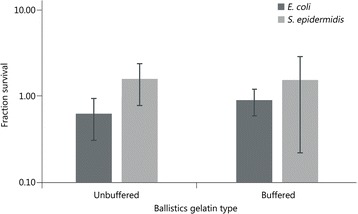
Fraction of survival of *E. coli* and *S. epidermidis* in solid gelatin after 60 min compared to time zero. No significant difference in survival was noted. The error bars represent the standard deviation

The effect of direct contact of the bacterial contaminants with solid gelatin was tested, i.e., the environment of the contaminants immediately after the projectile had deposited the contaminants in the wound track (Fig. 1), and prior to the processing of the simulated wound track. To encase the bacterial contaminants in the gelatin, a liquid culture was mixed with melted gelatin, and the gelatin was placed at 4 °C to solidify. Since the volumes used were quite small in comparison to the volume used to prepare the gelatin blocks, the solidification was complete in minutes. Both *E. coli* and the gram-positive skin bacterium *Staphylococcus epidermidis* were tested. Following incubation in the solid gelatin, the gelatin was re-melted, and bacterial survival was determined as described in the Methods section. For both contaminant organisms, there was no significant decrease in survival following this treatment compared to time zero (Fig. 6).

### Bacterial survival supported by buffering ballistics gelatin

Since direct contact with solid gelatin was not detrimental to bacterial survival, the possibility that the melted gelatin in buffer was bactericidal was considered by incubation in a 42 °C water bath. We found that gelatin prepared according to standard specifications with local tap water had an acidic pH of 5.1. To neutralize the gelatin and optimize bacterial survival, multiple microbiologically relevant phosphate buffers were used for gelatin preparation as well as for the recovery of the bacterial contaminants during melting of the gelatin. The pH of the melted gelatin as well as the gelatin/buffer mixtures was measured (Table 1). The PBS did not have a significant buffering effect on the ballistics gelatin; both RO water and PBS produced more acidic conditions than tap water alone. Buffering with M9, M63 and high pH phosphate buffer all increased the pH to be closer to neutrality.

Survival of the bacterial contaminants was tested in each of the gelatins prepared from different buffers, using the same buffer to melt the gelatin as would be used for the recovery of the contaminants from the permanent cavity of the wound track. Survival was also measured over the same time period in the buffer alone without gelatin to determine the effect of the 42 °C temperature required to melt the gelatin. Survival was measured over 40 min, which represents the maximum time required to process a wound track. Both *E. coli* and *S. epidermidis* were tested.

**Fig. 7:**
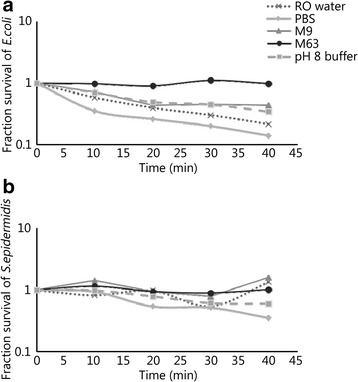
Fraction of survival compared to time zero with a gelatin “plug” and different buffers. **a** *E. coli.* Significantly different survival between M63 buffer (*P* < 0.005) and M9, PBS, pH 8 buffer, RO; **b** *S. epidermidis.* No significant difference in survival

Survival of *E. coli* was lower for both the RO water and PBS buffer/gelatin mixture over time, compared with M63, M9 and phosphate buffer pH 8 (Fig. 7a). *S. epidermidis,* on the other hand, was less sensitive to acidic conditions, and showed high survival in all the buffers with gelatin (Fig. 7b).

**Fig. 8:**
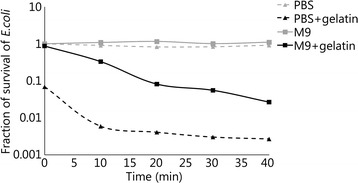
Fraction of survival of *E. coli* in buffer alone versus buffer and gelatin. Survival is calculated based on the initial survival of *E. coli* in its corresponding buffer alone. A significant difference in survival between PBS (buffer only) and PBS buffer plus ~ 1 ml of gelatin, and between M9 (buffer only) and M9 buffer plus 1 ml of gelatin (*P* < 0.005) was seen

Because the consistency of gelatin is vital to the reproducibility of contamination testing, and RO water may vary in content depending on the production apparatus, gelatin and buffers in which the RO water was replaced with commercially available molecular grade water (Milli-Q filtration unit, Millipore, Massachusetts, USA) were also prepared. An additional test was performed with M9 and PBS buffer, and gelatin prepared with molecular water, and the survival of *E. coli* in relation to initial survival in corresponding buffer only was measured (Fig. 8). Once again, buffer alone at 42 °C had no detrimental effect on survival. For the RO water buffers, the *E. coli* survived melted gelatin and M9 buffer better than in PBS; however, there was still a 10-fold reduction in survival after 20 min.

## Discussion

Infection resulting from both skin laceration and the environment through which a projectile has passed is a serious concern in a military scenario, in which access to immediate medical attention may be absent. In addition, the vacuum effect of the temporary cavity generated by the passage of a projectile as well as the closure of the wound track following penetration provide both the required inoculum as well as a potential breeding ground for severe infection. Furthermore, adequate wound healing is impeded when a bacterial infection is present [1]. Understanding both the bacterial distribution in a wound as well as the source of the contaminant from the surrounding environment are vital for debridement, treatment, and prevention. Collagen gelatin is a common biological tissue substitute used to study projectile perforation and bacterial distribution. While not a perfect representation of heterogeneous tissue, it is convenient and generates reproducible results in initial testing and modeling. Gelatin is translucent for use for high-speed video recording and is commonly used as a soft tissue surrogate in medical and forensic research. It has a low melting temperature (~ 42 °C), which is adequate for bacterial studies.

The current methods being used for bacterial distribution studies (see Fig. 1, for example) have demonstrated that bacteria on a surrogate skin will disperse throughout a simulated wound track when pierced by a projectile [3–5, 14–17]. However, these studies have shown inconsistent quantification and poor repeatability between parallel tests. Ideally, a bacterial distribution model would demonstrate reproducible results with contaminants representative of the environment under study (e.g., skin, clothing, soil, etc.). We have identified several aspects related to the contamination model that may not have been previously considered as areas that may impact bacterial distribution and survival in experiments.

The actual bacterial contaminant on the surrogate “skin” is the first factor that can have a significant impact on the results. The bacterial strain must be non-pathogenic to satisfy biosafety protocols. It would ideally be representative of its environment and/or easily distinguishable from other types of contamination that must be eliminated from the results. The normal skin bacterium *Staphylococcus epidermidis* is non-pathogenic and easy to grow. Alternatively, *Escherichia coli* is a common inhabitant of water contaminated with sewage, and has a broad array of inexpensive, available genetic tools that may be used to confer antibiotic resistance or color changes for the easy elimination of background contaminants.

Once the contaminant is selected, it must be loaded onto the skin surrogate. In many previous studies, the contaminant was merely swabbed or pipetted onto the surrogate and allowed to dry before mounting to the gelatin target [3–5, 14–17]. Using this technique, it is impossible to accurately predict how much bacteria is present at a specific site, nor is it possible to evenly distribute a known concentration of bacteria. Vacuum filtration is a novel application for generating a contamination model, and it has many advantages over previous methods. A filter paper of the appropriate pore size was chosen to capture all of the bacteria in a liquid sample, and the filter was large enough in diameter to account for some imprecision in the projectile impact location. Filtration of a homogenous liquid culture allows for the even distribution of cells across the entire diameter of the filter. It is also possible to precisely calculate the total number of viable cells on the filter paper, and change that number based on the experimental requirements. In this study, *E. coli* recovery from the filter paper was consistent across the entire area of the filter, even using different amounts of dilution buffer. The percentage of bacterial recovery versus the bacterial load, while low at 2%, was consistent and may be different when using alternative filters, so the recovery should be calibrated before a bacterial distribution study is performed. Finally, since drying the contamination model prior to mounting did not seem to affect bacterial recovery or survival, the “pre-loading” of several filters for multiple consecutive tests could be accomplished without concern about the effect on recovery or survival of the bacterial contaminants.

If conventional Hydrargyrum Medium-Arc Iodide (HMI) high-intensity lighting is being used to record a high-speed video of of bacterial distribution tests, prolonged exposure may have a negative effect on bacterial survival. In fact, high-intensity lights proved to be extremely bactericidal to the contamination model. Therefore, the use of the high-intensity lights should be strictly limited once the filter paper is placed on the ballistics gelatin target. High-intensity LED lights may be another alternative, because the heat generated and the UV radiation emitted are much lower. However, high-intensity LED lights may not provide the level of illumination required for the frame rates and shutter speeds used to record ballistic penetration events.

The final factors that were investigated focused on bacterial survival in the ballistics gelatin itself. After projectile penetration, the bacteria spends a relatively long time in the solid gelatin wound track before excision. Additionally, the gelatin must be melted and diluted so it can be plated, and the bacterial concentration calculated. It was important to understand if prolonged exposure to 42 °C in the buffer/gelatin solution was bactericidal. In solid gelatin, survival of both *E. coli* and *S. epidermidis* was stable for an hour, so the time to accomplish the “wound track” excision is not a major factor for survival. It was therefore somewhat surprising to discover that gelatin melted in buffer had a detrimental effect on bacterial survival, but further study revealed this may be at least partially due to the low pH of gelatin prepared in tap water. Microbiologically relevant neutral and slightly basic buffers allowed the pH of the gelatin to be neutralized and improved bacterial survival in buffer/gelatin at 42 °C. Interestingly, even though both *E. coli* and *S. epidermidis* were completely stable in buffer alone at 42 °C, there was still a significant loss with time in the buffer/gelatin mixtures. Therefore, these must be processed and plated as soon as the gelatin is dissolved.

The experiments were aimed primarily at optimizing the methods used in current bacterial distribution studies in ballistics gelatin targets shot with small caliber projectiles from a microbiological rather than an engineering perspective. The testing procedures and protocols for such studies must be as consistent and repeatable as possible to glean useful information that can be applied to the prevention and treatment of wound contamination and subsequent infection. Factors that can affect bacterial survival and quantification have been addressed to improve reproducibility in studies in which the projectile size and speed may vary. Such future studies will help predict the potential bacterial load delivered in a wound and may aid in devising immediate treatment plans for reducing infection from small, dispersive projectiles.

## Conclusion

One aspect of interest in the study of penetrating projectile wounds is how bacteria present in the skin and clothing are transported into the wound track and distributed along the permanent cavity. To explore this topic, different authors have proposed experiments involving ballistics gelatin targets and different bacterial contamination models. Although the proposed contamination models have been useful to show the presence of bacteria along the entire surrogate wound track as well as to highlight bacterial distribution differences due to changes in parameters such as projectile speed, difficulties have been encountered to accurately quantify the number of bacteria delivered as well as the presence of consistent bacterial contamination loads. Furthermore, aspects affecting bacterial survival during typical bacterial distribution studies have not been formally considered.

The results presented in this paper provide two important contributions. First, a contamination model that can provide a more even bacterial contamination was proposed and assessed. Second, potential factors affecting bacterial survival during typical bacterial distribution studies involving ballistics gelatin targets have been identified and explored in the context of the proposed contamination model. The relevance of the experiments proposed in the paper is that they can be easily adapted and used to evaluate the performance of other contamination models proposed in the future.

The use of a filter paper and vacuum filtration to produce the source for bacterial contamination proved to be an effective way to have a consistent and uniform bacterial distribution in an area large enough to compensate for typical variations in the projectile impact location encountered during ballistic testing. Furthermore, using such an approach, it is feasible to adjust the bacterial concentration per unit of area to the contamination level required for a particular study.

In addition to a bacterial contamination source such as the one described above, to obtain repeatable quantitative results in bacterial distribution studies, it is necessary to identify and mitigate any factors that can adversely affect bacterial survival during the tests. Those factors can vary depending on the specific bacteria selected for the contamination model and the testing protocol used. For *E. coli*, we showed that it is necessary to prepare the ballistics gelatin using properly buffered solutions instead of regular tap water to avoid pH levels that can have a negative impact on bacterial survival. Furthermore, we showed that exposure to high-intensity lights was highly bactericidal, even for short periods of time. Since recording high speed video during tests is required to capture the transient dynamic behavior of the ballistics gelatin during the formation and collapse of the temporary cavity, it is important to minimize the exposure time to the high-intensity lights as much as possible and to use the same exposure time in all the tests conducted to avoid variations in the bacterial counts due to this factor, which is unrelated to the physics of the projectile penetration event.

## Abbreviations

CFU: Colony forming units
*E. coli*: *Escherichia coli*
PBS: Phosphate buffered saline
RO: Reverse osmosis
*S. epidermidis*: *Staphylococcus epidermidis*
